# Stability of numerous novel potassium chlorides at high pressure

**DOI:** 10.1038/srep26265

**Published:** 2016-05-23

**Authors:** Weiwei Zhang, Artem R. Oganov, Qiang Zhu, Sergey S. Lobanov, Elissaios Stavrou, Alexander F. Goncharov

**Affiliations:** 1 Department of Applied Physics, China Agricultural University, Beijing, 100080, China; 2 Department of Geosciences, Center for Materials by Design, and Institute for Advanced Computational Science, State University of New York, Stony Brook, New York 11794-2100, USA; 3 Skolkovo Institute of Science and Technology, Skolkovo Innovation Center, 3 Nobel St., Moscow 143026, Russia; 4 Moscow Institute of Physics and Technology, 9 Institutskiy Lane, Dolgoprudny city, Moscow Region 141700, Russia; 5 Northwestern Polytechnical University, Xi’an 710072, China; 6 Geophysical Laboratory, Carnegie Institution of Washington, 5251 Broad Branch Road, Washington, D.C. 20015, USA; 7 V.S. Sobolev Institute of Geology and Mineralogy, SB RAS, 3 Pr. Ac. Koptyga, Novosibirsk 630090, Russia; 8 Lawrence Livermore National Laboratory, Physical and Life Sciences Directorate, P.O. Box 808 L-350, Livermore, California 94550, USA; 9 Key Laboratory of Materials Physics, Institute of Solid State Physics, CAS, Hefei, 230031, China; 10 University of Science and Technology of China, Hefei, 230026, China

## Abstract

K-Cl is a simple system displaying all four main types of bonding, as it contains (i) metallic potassium, (ii) elemental chlorine made of covalently bonded Cl_2_ molecules held together by van der Waals forces, and (iii) an archetypal ionic compound KCl. The charge balance rule, assigning classical charges of “+1” to K and “−1” to Cl, predicts that no compounds other than KCl are possible. However, our quantum-mechanical variable-composition evolutionary simulations predict an extremely complex phase diagram, with new thermodynamically stable compounds K_3_Cl, K_2_Cl, K_3_Cl_2_, K_4_Cl_3_, K_5_Cl_4_, K_3_Cl_5_, KCl_3_ and KCl_7_. Of particular interest are 2D-metallic homologs K_n+1_Cl_n_, the presence of positively charged Cl atoms in KCl_7_, and the predicted stability of KCl_3_ already at nearly ambient pressures at zero Kelvin. We have synthesized cubic 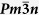 *-*KCl_3_ at 40–70 GPa and trigonal 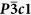 -KCl_3_ at 20–40 GPa in a laser-heated diamond anvil cell (DAC) at temperature exceeding 2000 K from KCl and Cl_2_. These phases were identified using *in situ* synchrotron X-ray diffraction and Raman spectroscopy. Upon unloading to 10 GPa, 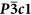 -KCl_3_ transforms to a yet unknown structure before final decomposition to KCl and Cl_2_ at near-ambient conditions.

Recent *ab initio* calculations predicted the formation of unexpected novel high-pressure compounds in several simple systems, such as Li (Na)-H^1^,^2^ Mg-O^3^, and Na-Cl^4^. These systems were subsequently explored experimentally: while so far the predictions have not been verified for Li-H^5^, for Na-Cl and Mg-O, the predicted compounds (NaCl_3_, Na_3_Cl, and MgO_2_) have been confirmed experimentally^4^,^6^, while more stable NaH_x_ compounds than originally predicted by Zurek *et al*.^2^ have been synthesized and theoretically verified^7^, revealing dramatic changes of chemistry under pressure. Here we study K-Cl, a system closely related to Na-Cl and find even richer chemistry and new phenomena.

The only known potassium chloride, KCl, has been extensively studied under pressure, both experimentally^8^,^9^,^10^ and using *ab initio* simulations^11^,^12^,^13^. Two crystal structures are known for KCl: the rocksalt-type (B1) structure and cesium chloride-type (B2) structure, the latter becoming stable at ~2 GPa. The same transition occurs in NaCl, but at a much higher pressure of 30 GPa^14^,^15^, reflecting the general tendency for phase transitions to occur at lower pressures for compounds of heavier elements. Yet, as we find, the K-Cl system has a much richer chemistry than Na-Cl. Here we study the K-Cl system using the quantum-mechanical variable-composition evolutionary structure prediction methodology USPEX^16^,^17^,^18^,^19^, searching for stable compounds and their corresponding crystal structures (see Methods). In each of these calculations, all possible chemical compositions were allowed with up to 16 atoms in the unit cell, and calculations were performed at pressures of 1 atm, 10 GPa, 35 GPa, 50 GPa, 100 GPa, 150 GPa, 200 GPa, 250 GPa and 300 GPa. Theoretical predictions were successfully verified by experimental synthesis of two KCl_3_ polymorphs in a laser-heated diamond anvil cell (DAC).

## Results and Discussion

**Figure 1:**
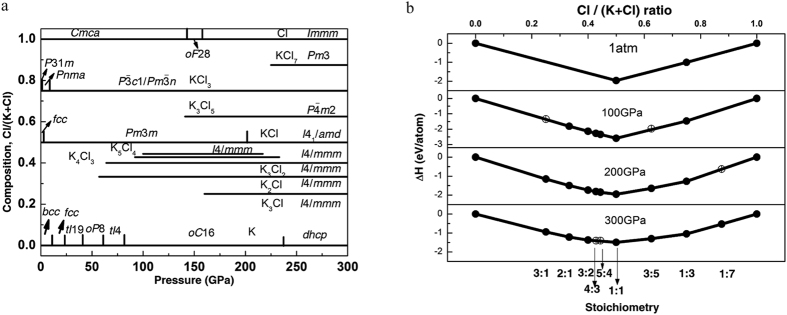
Stability of new potassium chlorides: (**a**) Pressure-composition phase diagram of the K-Cl system. (**b**) Convex hull diagrams for the K-Cl system at selected pressures. Solid circles represent stable compounds; open circles - metastable ones.

The pressure-composition phase diagram (see Fig. 1, Supplementary Fig. S1) predicted in our calculations contains a surprisingly large number of new stable compounds (see Supplementary Table S1). By thermodynamically stable we mean a compound which is more stable than any isochemical mixture of the elements or other compounds – this definition leads to the convex hull construction shown in Fig. 1b. The dynamical stability of the newly predicted phases was confirmed by phonon calculations (see Supplementary Fig. S2).

To verify these predictions, we performed high-pressure experiments on the K-Cl system in a laser-heated DAC up to 70 GPa in the presence of excess chlorine. We specifically targeted synthesis of KCl_3_, which was predicted to become stable at the lowest pressures. The reaction products were examined by visual observations (see Supplementary Fig. S3), Raman confocal spectroscopy, and synchrotron x-ray diffraction (XRD) probes at room temperature. Combining experimental and theoretical approach was critical to refine the K-Cl phase diagram as several KCl_3_ phases showed competing enthalpies in the 0–30 GPa pressure range.

**Figure 2:**
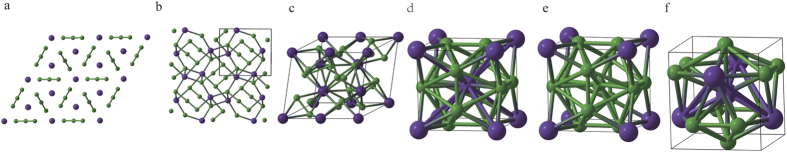
Crystal structures of (**a**) *P*31*m*-KCl_3_ at 1atm. (**b**) *Pnma*-KCl_3_ at 5 GPa. (**c**) *P *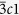-KCl_3_ at 20 GPa. (**d**) 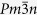
*-*KCl_3_ at 240 GPa. (**e**) *Pm*3*-*KCl_7_ at 240 GPa. (**f**) *P*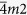-K_3_Cl_5_ at 240 GPa.

The phase diagram shows that KCl remains stable in the whole pressure range investigated here, but many new compounds become stable at elevated pressures. Perhaps most unexpected is the prediction that KCl_3_ is stable already at 1 atm and 0 K. The structure belongs to *P*31*m* space group with 3 formula units (f.u.) in the unit cell (Fig. 2a), and contains exotic trichloride-ions Cl_3_^−^. The *P*31*m* phase is a semiconductor, with a DFT band gap of 2.60 eV. The first phase transition is to the *Pnma* structure at 1.3 GPa. *Pnma* structure has 4 f.u. in the unit cell and also contains trichloride-ions (Fig. 2b). Bader analysis gives the charge configuration K^+0.83^[Cl^−0.28^Cl^−0.04^Cl^−0.51^]^−0.83^, nearly the same as for *Pnma*-NaCl_3_ [ref. 4]. [Cl_3_]^−^ ion is an isoelectronic analogue of the well-known triiodide-ion [I_3_]^−^ (for example, compound KI_3_ is well known), Br_3_^−^ and ClICl^−^ ions, and can be also related to the known [Li_3_]^−^ [ref. 20] and hypothetical [H_3_]^−^ [ref. 5] ions. At 9.3 GPa, *P *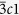 structure of KCl_3_ with 6 f.u. (Fig. 2c) in the unit cell becomes stable. The DFT band gap of *P *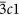-KCl_3_ is 1.78 eV at 20 GPa. Interestingly, at P > 160 GPa, *P *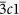-KCl_3_ turns metallic due to the band gap closure. Metallic 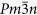-KCl_3_, isostructural with stable *P *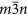-NaCl_3_, is also energetically competitive under pressure. K_3_Cl_5_ and KCl_7_ become stable at pressure above 140 GPa and 225 GPa, respectively.

**Figure 3:**
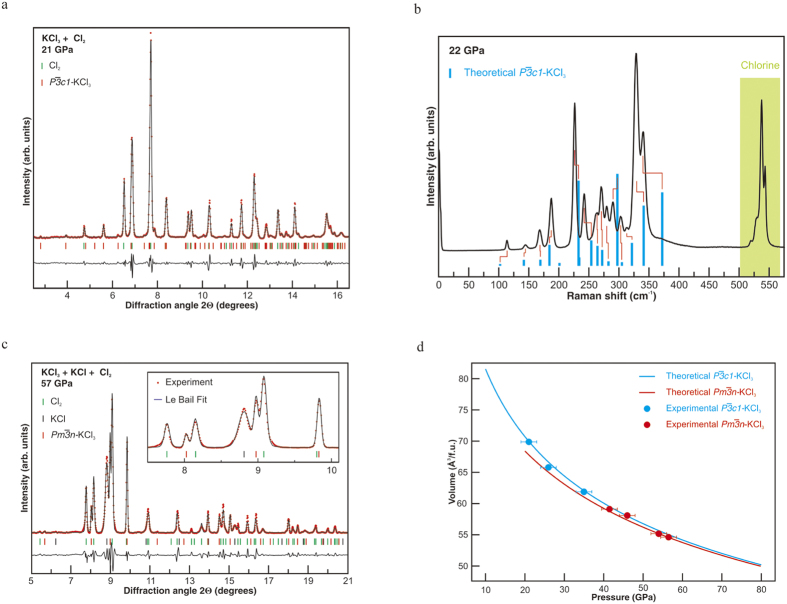
Experimental evidence for KCl_3_: (**a**) XRD pattern of *P *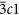-KCl_3_ and Cl_2_ at 21 GPa. (**b**) Raman spectrum of *P *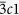-KCl_3_ and Cl_2_ at 22 GPa. Blue bars show computed spectral positions and intensities; Red ticks represent tentative assignment of the theoretically predicted Raman modes with the experimental data. (**c**) XRD pattern of 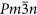-KCl_3_, KCl, and Cl_2_ at 57 GPa. (**d**) Experimental and theoretical pressure-volume equations of state of *P *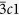 and 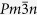-KCl_3_. In (**a**,**c**) black lines show the intensity difference (I_obs_ − I_calc_), Le Bail refinement residuals are R_wp_ = 0.139 and R_exp_ = 0.096 in (**a**) and R_wp_ = 0.233 and R_exp_ = 0.151 in (**c**). X-ray wavelengths are 0.3100 Ǻ in (**a**) and 0.3344 Ǻ in (**c**).

We synthesized KCl_3_ at elevated pressures and temperatures at conditions of excess of Cl_2_. To overcome the kinetic barriers, the reagents were laser-heated above 2000 K. The temperature was determined radiometrically. This heating procedure also promotes better mixing of reagents as chlorine melts and becomes highly diffusive. Pressures in excess of 20 GPa were necessary to initiate a chemical reaction between KCl and Cl_2_. A set of new Bragg peaks was observed after laser heating at 20–40 GPa with intensities and angular positions in agreement with *P *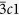-KCl_3_ (Fig. 3a). Rietveld refinement, however, was not possible because of the apparent texturing of new reflections (see Supplementary Fig. S4). A rich Raman spectrum, with at least 15 peaks (Fig. 3b), was observed for the synthesized compound, which is consistent with group theory allowing 16 Raman active modes (Г = 5A_1g_ + 11 E_g_) for *P *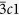-KCl_3_. Likewise, Raman shift of the experimentally observed bands agrees well with that computed for *P *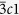-KCl_3_ at corresponding pressures (see Supplementary Fig. S5). Typical agreement of vibrational frequencies from DFT and experiment for materials with well-established structure is up to 10%, e.g. ref. 21. Therefore, experiments confirm that *P* 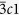-KCl_3_ is the most stable phase in the 20–40 GPa pressure range.

XRD of quenched samples prepared at P > 35–40 GPa shows a mixture of two space-separated phases of KCl_3_: *P *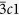 and 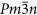. Larger yields of the 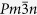 phase were achieved at P > 50–60 GPa (Fig. 3c) in qualitative agreement with theoretical predictions showing that the energy difference between *P*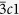 and 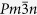-KCl_3_ decreases with increasing pressure. We could only use the Le Bail refinement of 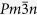-KCl_3_ because of the spotty character of XRD images (see Supplementary Fig. S6). The agreement between the experimentally measured and computed equations of state of KCl_3_ (Fig. 3d) further validates theoretical predictions. It is remarkable that in a number of experiments KCl reacted completely, forming KCl_3_, with the only remaining material in the probed area being *Cmca-*chlorine, which was easily identified based on experimental^22^ and our theoretically calculated lattice parameters.

On decompression to below 10 GPa, Raman bands of *P *
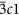
*-*KCl_3_ disappeared completely, while new strong pressure-dependent bands appeared near 450 cm^−1^ (see Supplementary Fig. S7). We tentatively assigned these bands to stretching vibrations of the linear Cl_3_^−^ ions^23^. Changes in XRD also suggest a phase transition, although the quality of the diffraction pattern was not sufficient to index new peaks and pinpoint the structure. At room temperature, this new phase becomes unstable below 2 GPa: Raman spectroscopy, X-ray diffraction and visual observations showed only the presence of KCl and Cl_2_ in the decompressed sample cavity.

**Figure 4:**
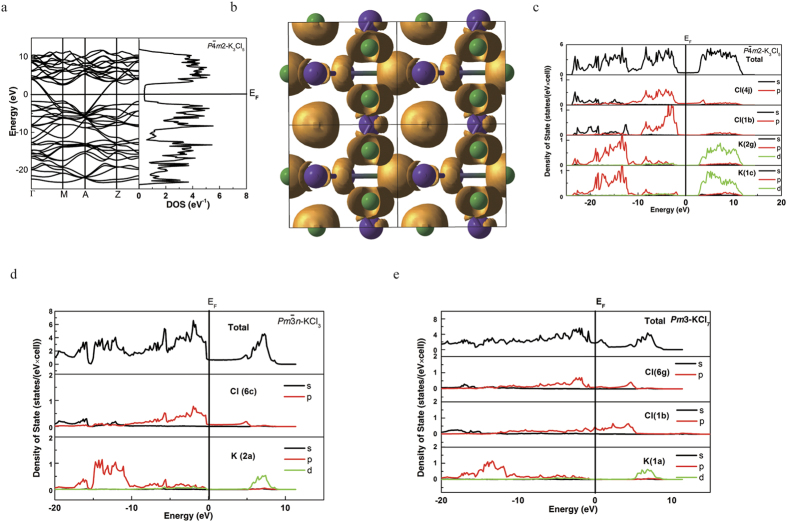
Electronic structure: (**a**) band structure and electronic density of states of *P*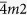-K_3_Cl_5_ at 240 GPa. (**b**) electron localization function of *P*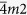-K_3_Cl_5_ at 240 GPa with isosurface ELF = 0.77. (**c**) total and atom-projected densities of states of *P*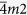-K_3_Cl_5_. (**d**) total and atom-projected densities of states of 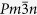-KCl_3_. (**e**) total and atom-projected densities of states of *Pm*3-KCl_7_ at 240 GPa.

In the K-Cl system, in contrast with Na-Cl, there is yet another chlorine-rich phase, *P *
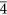
*m*2-K_3_Cl_5_, which has a pseudocubic cell with 1 formula unit. The K atom in the center of the unit cell is surrounded by 4 K atoms and 10 Cl atoms, together forming a bicapped hexagonal antiprism (Fig. 2f). The electronic structure (Fig. 4a,c) shows that it is a poor metal with a deep pseudogap of width ~4.6 eV at 240 GPa. In Fig. 4, we compare the total and atom-projected electronic densities of states of *P *
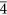
*m*2-K_3_Cl_5_, 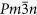
*-*KCl_3_ and *Pm*3*-*KCl_7_. All these phases are poor metals with pronounced pseudogaps at the Fermi level, implying electronic stabilization. The main contribution at the Fermi level comes from chlorine atoms, and one can observe that different chlorine sites play very different roles – for example, in *P*
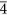
*m*2-K_3_Cl_5_ only p-orbitals of Cl (4j) contribute at the Fermi level, and are thus responsible for its metallicity. Due to excess of chlorine atoms, which act as electron acceptors, 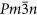
*-*KCl_3_ has DOS similar to p-type semiconductors. The central, positively charged Cl (1b) donating electrons to the system in *Pm3-*KCl_7_, makes the DOS at the Fermi level in KCl_7_ much higher than that in *P*
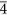
*m*2-K_3_Cl_5_ and 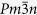
*-*KCl_3_. Distributions of valence electron localization function (ELF, e.g., Fig. 4b) also show that crystallographically inequivalent Cl atoms have very different ELF distributions – from spherical (around atoms with the most negative Bader charge, indicating a closed-shell configuration, and also around the positively charged Cl atom in KCl_7_) to toroidal (around atoms with small negative charges).

Comparing Bader charges of K-Cl phases (see Supplementary Table S2) with those of Na-Cl phases, we see higher charges on Na atoms in *Pnma*-NaCl_3_, 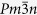-NaCl_3_ and *Pm*3-NaCl_7_ (about +0.8) than in their K-counterparts (about +0.65). This is counterintuitive, but consistent with our finding^24^ that under pressure K has higher electronegativity and lower reactivity than Na, due to the well-known s → d electronic transition in K atoms under pressure. Related to this is the observation that the depth of the convex hull (i.e. the enthalpy of formation of KCl or NaCl) in the K-Cl system (Fig. 1) changes from −2.9 eV/atom at 40 GPa to −1.5 eV/atom at 300 GPa, whereas for the Na-Cl system^3^, it changes from −2.5 eV/atom at 40 GPa to −2.9 eV/atom at 300 GPa.

**Figure 5:**
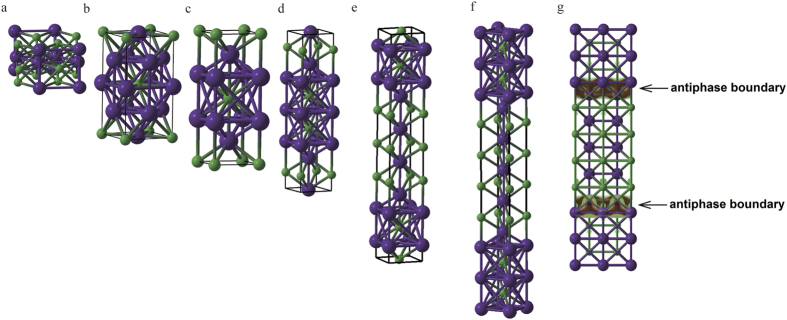
Crystal structures of (**a**) high-pressure *I*4_1_*/amd*-KCl, (**b**) fcc-derived *I*4/*mmm*-K_3_Cl, and bcc-derived K_n+1_Cl_n_ homologs: (**c**) *I*4/*mmm*-K_2_Cl, (**d**) *I*4*/mmm*-K_3_Cl_2_, (**e**) *I*4*/mmm*-K_4_Cl_3_, (**f**) *I*4*/mmm*-K_5_Cl_4_. (**g**) Spatial distribution of electrons (shown by isosurfaces and density contours) at the Fermi level in *I*4*/mmm*-K_5_Cl_4_, showing clear 2D-metallic character.

In the studied pressure range, besides the known B1 and B2 phases, we find a new phase of KCl: *I*4_1_*/amd*–KCl, stable above 201 GPa, shown in Fig. 5a. This structure is a derivative of the fcc structure. Figure 5b shows K_3_Cl, the other fcc-derived superstructure compound stable in the K-Cl system (above 149 GPa) – square planar layers with stoichiometry KCl alternate with similar layers of stoichiometry K_2_ along the *c*-axis, leading to the total stoichiometry K_3_Cl. These two compounds can be described as fcc-based homologs.

There is another interesting and surprisingly rich class of phases, K_n+1_Cl_n_ homologs (*n* = 2, 3, 4 were found in our calculations, but we cannot exclude the possibility of even higher homologs) based on the B2 structure and shown in Fig. 5c–g. These have (2*n* + 1) layers along the *c*-axis, with extra K-layer serving as an antiphase boundary between B2-structured domains. All these phases have the same space group *I*4*/mmm,* and similar interatomic distances, all of them are poor metals, due to the excess of electron-donating K atoms, analogous to n-type semiconductors (see Supplementary Figs S8 and S9), and display a two-dimensional metallic character. It is surprising that phases with different *n*, while being structurally so similar, have quite different stability fields: e.g., K_2_Cl is stable at pressures above 56 GPa, whereas K_5_Cl_4_ is stable above 100 GPa. Interestingly, mobile electrons are observed only at the antiphase boundaries, whereas regions between them are insulating (Fig. 5g). These antiphase boundaries may be created as metastable growth defects also at lower pressures, with the promise of new electronic materials.

In summary, for a seemingly simple K-Cl system our calculations predict an extremely unusual behavior. Already at near-ambient conditions we have predicted and experimentally confirmed the existence of a new insulating compound KCl_3_, which has not been observed before. As pressure increases, a surprisingly large number of thermodynamically stable phases become stable: (1) Cl-rich metallic phases (KCl_7_, K_3_Cl_5_, and a metallic form of KCl_3_) with high coordination numbers (12–14), (2) fcc-superstructures (insulating *I*4_1_*/amd-*KCl and metallic K_3_Cl), (3) layered B2-superstructures with compositions K_*n*+1_Cl_*n*_ (*n* = 3, 4, 5) and two-dimensional electronic conductivity. KCl_3_ decomposes into KCl and Cl_2_ at room temperature at pressures below 2 GPa and probably is stable at zero pressure and low temperatures, as suggested by theory. What was considered as an ultimately simple chemical system, upon careful theoretical and experimental study turned out to be a very rich system with novel physics and chemistry. Revisiting other simple systems may result in the formulation of new chemical principles that could be used for the discovery of novel materials and phenomena.

## Methods

### Theory

Structure/composition predictions were done using the USPEX code^16^,^17^,^18^ in the variable-composition mode^19^. The first generation of structures was produced randomly and the subsequent generations were obtained by applying heredity, transmutation, softmutation, and lattice mutation operations, with probabilities of 60%, 10%, 20% and 10%, respectively. 60% fittest non-identical structures of each generation were used to produce the next generation. 20% new random symmetric structures were also added in each generation. All structures were relaxed using density functional theory (DFT) calculations at the generalized gradient approximation level of theory, with the Perdew-Burke-Ernzerhof (PBE)^25^ exchange-correlation functional, as implemented in the VASP code^26^. We used the all-electron projector augmented wave (PAW)^27^ with K [3s^2^3p^6^4s^1^], Cl [2s^2^2p^4^] cores (core radii 2.20 a.u. and 1.50 a.u., respectively) and plane-wave basis sets with the 500 eV cutoff, and dense Monkhorst-Pack meshes with resolution better than 2π × 0.05 Ǻ^−1^. We used the normalized enthalpy of formation as fitness and visualized crystal structures and electron density distributions using the STM4 package^28^. Having identified the most stable compositions and structures, we relaxed them at pressures between 1 atm and 300 GPa using very accurate Brillouin zone sampling (Monkhorst-Pack meshes with resolution of better than 2π × 0.03 Ǻ^−1^).

### Experiments

We used symmetrical diamond anvil cells (DAC) to generate static pressures up to 70 GPa. The diamond culet size was 300 μm. Sample chambers were created by laser drilling of 70–80 μm holes in Re gaskets (40 μm thick). KCl platelets (8–15 μm thick), were made by squeezing KCl single crystals (which were dried out at 130 °C) and cleaved to match the dimensions of the gasket hole. Immediately after the cleaving, KCl platelets were stacked at an angle preserving empty space (5–15 μm) between the platelets for chlorine condensation in the gap. Subsequently, DACs were cooled to 77 K with liquid nitrogen in a nitrogen-purged glove box. Chlorine gas (Linde Group, >99.8%) was injected into the gasket cavity from a capillary. Finally, the DAC was closed and brought to high pressure while still at cryogenic temperatures.

Upon compression, chlorine becomes optically opaque and can be heated directly by a 1064 nm fiber laser. Double-sided laser heating experiments were performed at GeoSoilEnviroCARS (APS, Chicago) and Extreme Conditions Beamline P02.2 at DESY (Germany). Temperature was measured spectroradiometrically. XRD data were collected both at high temperature and from quenched samples. We used a 5 μm x-ray beam to detect phase transformations (chlorine melting, KCl3 synthesis) at high temperature, while 2–3 μm beam was used to map quenched samples.

Raman radiation was excited using either 488 or 532 nm lines of a solid state laser. The laser spot on the sample was focused to 4 μm. Raman spectra were analyzed with an imaging spectrograph equipped with a charge-coupled device (CCD). The spectral resolution was 4 cm^−1^.

## Additional Information

**How to cite this article**: Zhang, W. *et al*. Stability of numerous novel potassium chlorides at high pressure. *Sci. Rep.* **6**, 26265; doi: 10.1038/srep26265 (2016).

## Supplementary Material

Supplementary Information
