# Supplementary material for: Stability of numerous novel potassium chlorides at high pressure
Source: Sci Rep. 2016 May 23;6:26265. doi: 10.1038/srep26265 (PMC4876327; doi:10.1038/srep26265)
Supplement: Supplementary Information [file srep26265-s1.pdf]

Supplementary Information for:

Stability of numerous novel potassium chlorides at high pressure

Weiwei Zhang[1, 2 †], Artem R. Oganov[2-5, †], Qiang Zhu[2], Sergey S. Lobanov[6, 7], Elissaios Stavrou[6], Alexander F. Goncharov[6, 8, 9]

Figure S1 to S9, Table S1 to S2.

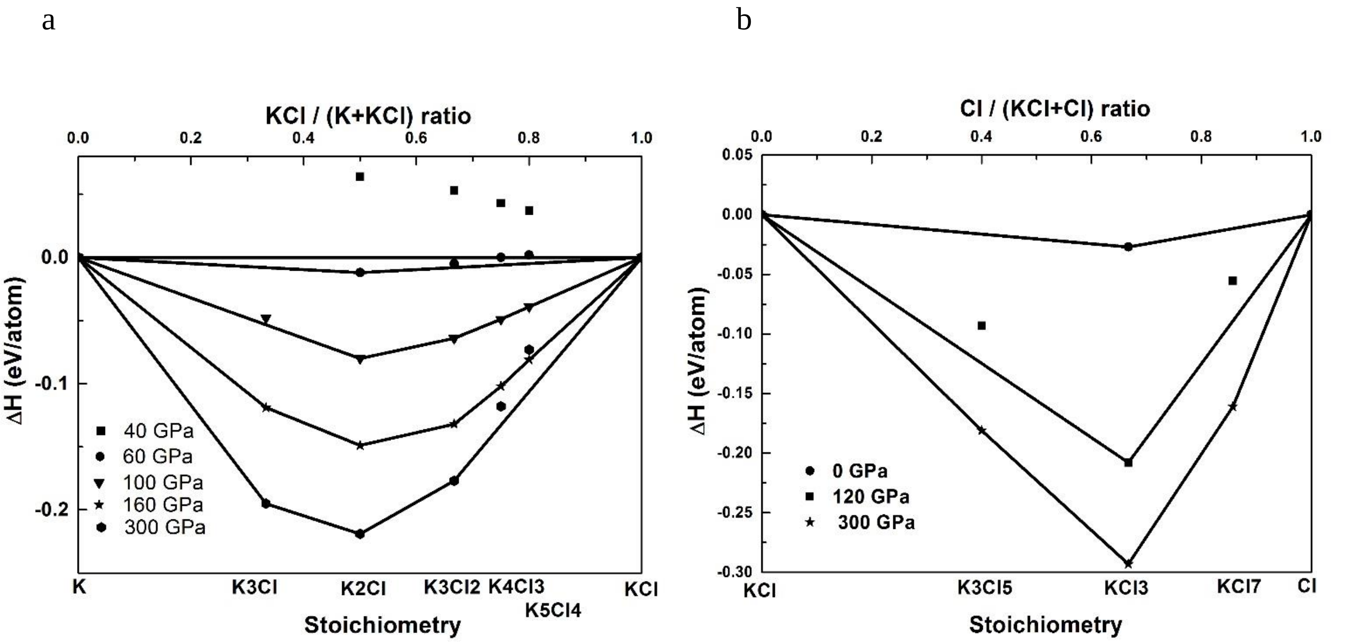

Supplementary Figure S1. Convex hull diagrams for (a) K-KCl system and (b) KCl-Cl system.

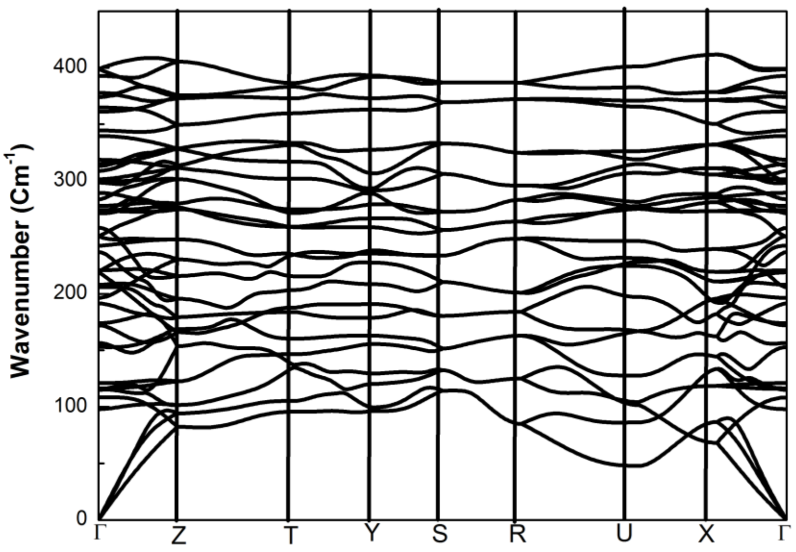

Supplementary Figure S2. Phonon dispersion curves of *Pnma*- KCl_3 at 30 GPa. Such calculations were done for all predicted structures to ensure their dynamical stability.

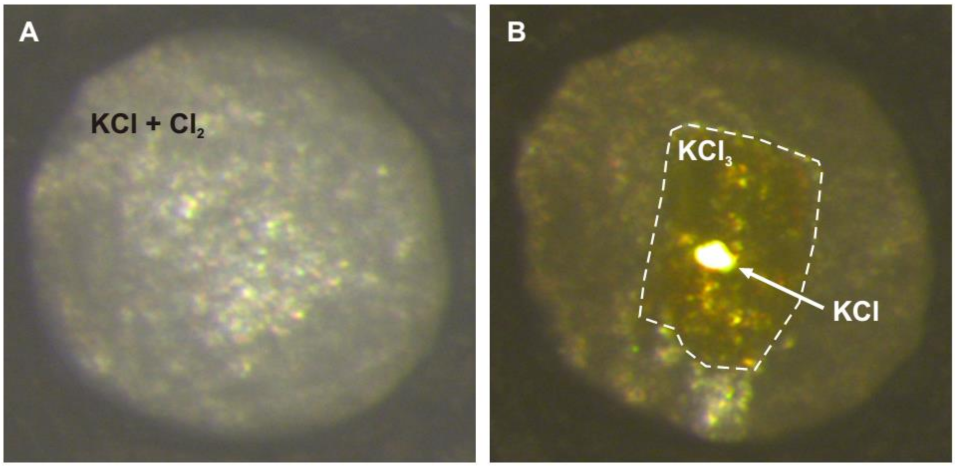

Supplementary Figure S3. Microphotograph of KCl + Cl_2 sample in the DAC cavity before (**A**) and after (**B**) laser heating at 25 GPa. In (**B**), the formation of new microcrystalline material is seen in the central area (marked by a white dashed line), which is surrounded by brownish KCl + Cl_2 unreacted sample. Transparent (bright white) spot in the center is pure KCl indicating that Cl_2 is consumed in the chemical reaction.

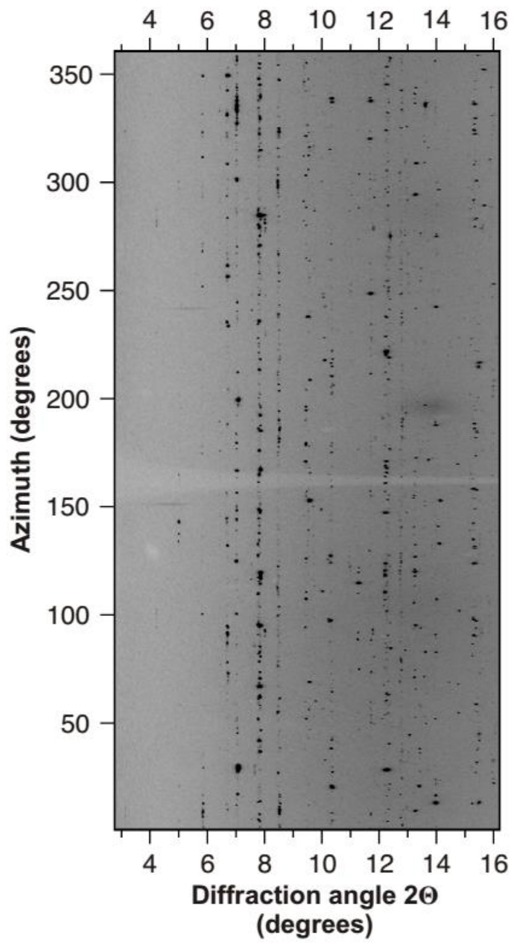

Supplementary Figure S4. Caked X-ray diffraction image of $P\bar{3}c1$-KCl_3 and Cl_2 at 21 GPa corresponding to Fig. 3a. The highly textured pattern of KCl_3 precluded Rietveld refinement. The wavelength is 0.3100 Å.

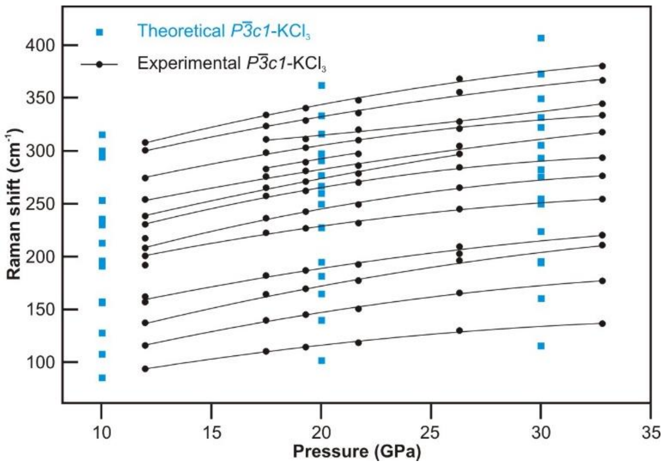

Supplementary Figure S5. Pressure dependence of the experimentally observed (black circles) vs theoretically computed $P\bar{3}c1$-KCl_3 (blue rectangles) Raman bands. Black curves are quadratic fits to the experimentally observed Raman frequencies.

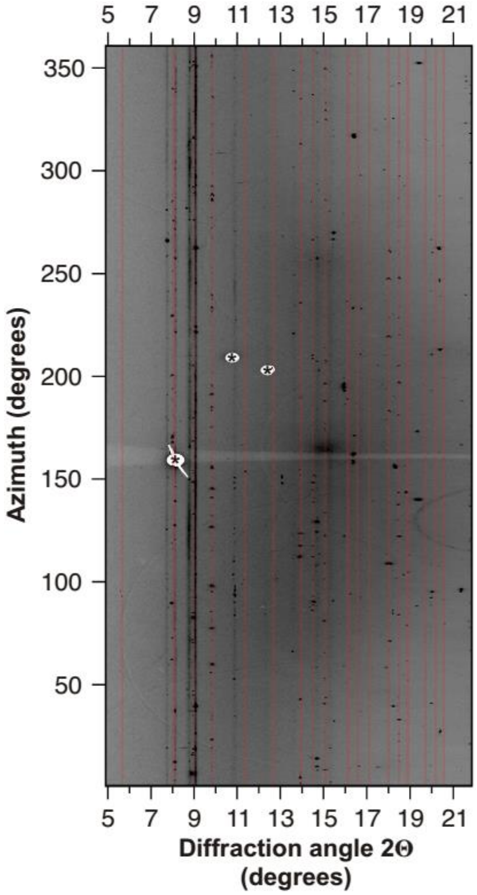

Supplementary Figure S6. Caked X-ray diffraction image of $Pm\bar{3}n$-KCl_3, KCl, and Cl_2 at 57 GPa corresponding to Fig. 3c. KCl and Cl_2 form quasi-continuous diffraction lines, whereas $Pm\bar{3}n$-KCl_3 shows a highly textured pattern (red lines). White areas marked with an asterisk mask saturated areas of the imaging plate. The wavelength is 0.3344 Å.

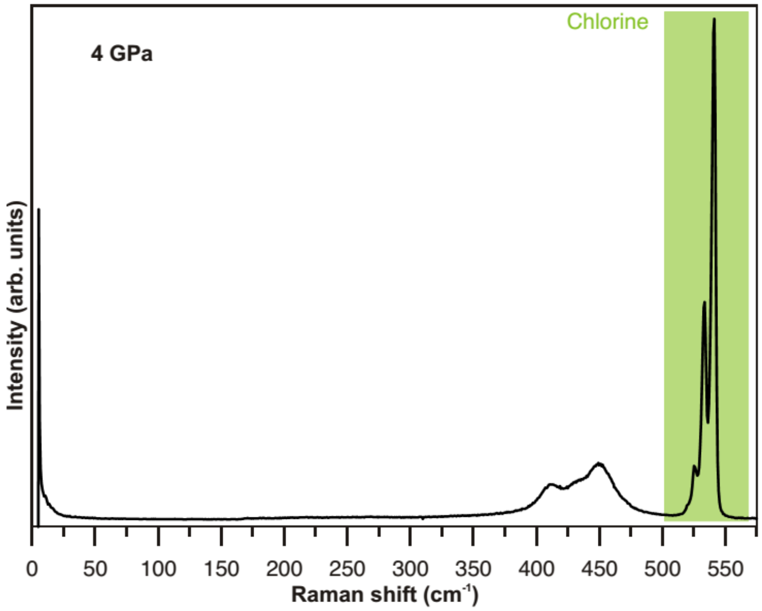

Supplementary Figure S7. Raman spectrum of a yet unknown K-Cl compound appearing on decompression to P < 10 GPa at 300 K. Chlorine peaks are in the shaded green area.

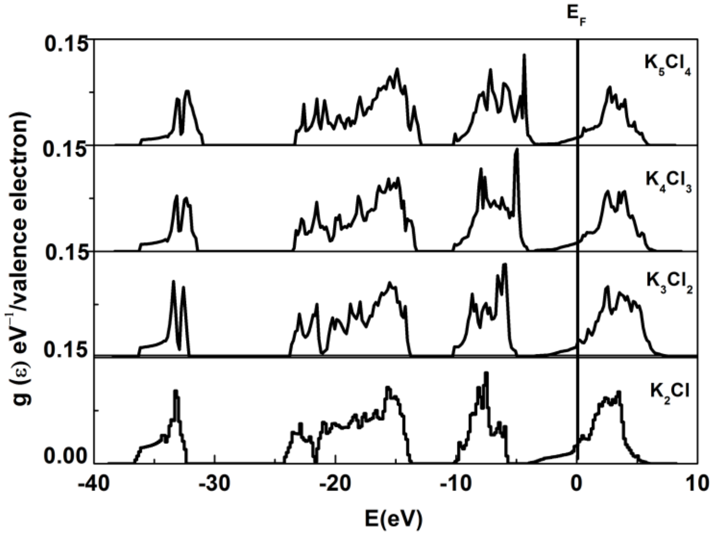

Supplementary Figure S8. The electronic density of states of layered $K_{n+1}Cl_n$ homologs at 200 GPa.

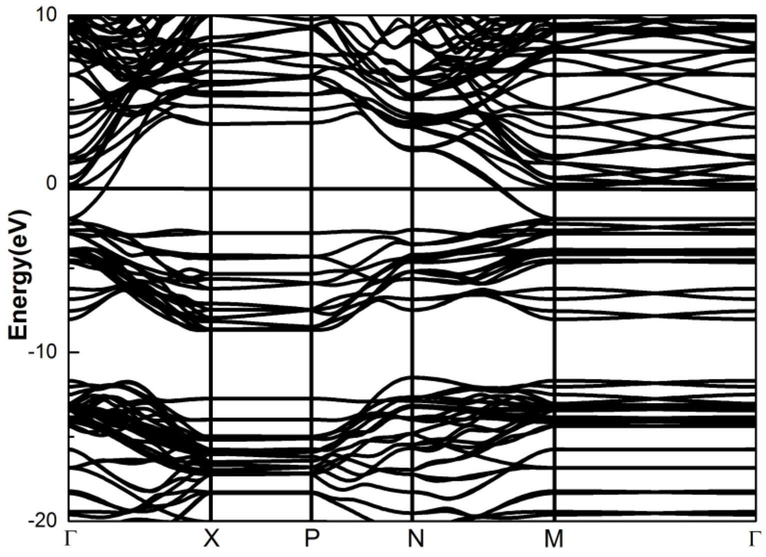

Supplementary Figure S9. Band structure of *I4/mmm*-K_5Cl_4 at 200 GPa.

Supplementary Table S1. Structure information and corresponding stability pressure regions for new compounds

Class of phases	Composition	Pressure range of stability	Space group	Cell parameters and atomic coordinates	
Insulating KCl	post-B2-KCl	201-300	$I4_1/amd$	*a* = 3.368 Å<br>*c* = 6.955 Å<br>(220 GPa)	K1 4a (0.000, 0.250, 0.875)<br>Cl1 4b (0.000, 0.750, 0.625)
Insulating Cl-rich phase	KCl_3	0-1.3	*P*31*m*	*a* = 8.587 Å<br>*c* = 6.206 Å（1atm)	K1 1a (0.000, 0.000, 0.504)<br>K2 2b (0.333, 0.667, 0.000)<br>Cl1 3c (0.667, 0.667, 0.251)<br>Cl2 3c (0.000, 0.512, 0.528)<br>Cl3 3c (0.000, 0.693, 0.805)
	KCl_3	1.3-9.3	*Pnma*	*a* = 8.708 Å<br>*b* = 5.426 Å<br>*c* = 7.946 Å<br>(5 GPa)	K1 4c (x, 0.250, z)<br>x=0.173, z= 0.464<br>Cl1 4c (x, 0.250, z)<br>x = 0.933, z = 0.769<br>Cl2 4c (x, 0.250, z)<br>x = 0.622, z = 0.552<br>Cl3 4c (x, 0.250, z)<br>x = 0.838, z = 0.360
Insulating and metallic Cl-rich phase	KCl_3	9.3-300	$P\bar{3}c1$	*a* = 7.347 Å<br>*c* = 9.059 Å<br>(20 GPa)	K1 2b (0.000, 0.000, 0.000)<br>K2 4d (0.333, 0.667, z)<br>z = 0.143<br>Cl1 6f (x, x, 0.250)<br>x = 0.260<br>Cl2 12g (x, y, z)<br>x=0.289, y=0.403,z= 0.897
Metallic Cl-rich phases	KCl_3	34-300	*Pm*3*n*	*a* = 4.169 Å<br>(240 GPa)	K 2a (0.000, 0.000, 0.000)<br>Cl 6c (0.000, 0.500, 0.250)
	KCl_7	225-300	*Pm*3	*a* = 4.123 Å<br>(240 GPa)	K 1a (0.000, 0.000, 0.000)<br>Cl1 1b (0.500, 0.500, 0.500)<br>Cl2 6g (x, 0.500, 0.000)<br>x=0.745
	K_3Cl_5	142-300	$P\bar{4}m2$	*a* = 4.136 Å<br>*c* = 4.361 Å<br>(240 GPa)	K1 1c (0.500, 0.500 ,0.500)<br>K2 2g (0.500, 0.000, -z)<br>z=-0.245<br>Cl1 1b (0.500, 0.500, 0.000)<br>Cl2 4j (x, 0.000, z)<br>x=0.252, z=0.747
Metallic $K_{n+1}Cl_n$, B2-layered superstructures	K_2Cl	56-300	*I*4/*mmm*	*a* = 2.749 Å<br>*c* = 7.587 Å<br>(200 GPa)	K1 4e (0.000, 0.000, z)<br>z=0.330<br>Cl 2a (0.000, 0.000, 0.000)
	K_3Cl_2	80-300	*I*4/*mmm*	*a* = 2.801 Å<br>*c* = 12.806 Å<br>(200 GPa)	K1 2b (0.500, 0.500, 0.000)<br>K2 4e (0.500, 0.500, z)<br>z = 0.798<br>Cl 4e (0.000, 0.000, z)<br>z = 0.897
	K_4Cl_3	62-264	*I*4/*mmm*	*a* = 2.791 Å<br>*c* =18.190 Å<br>(200 GPa)	K1 4e (0.000, 0.000, z)<br>z=0.072<br>K2 4e (0.000, 0.000, z)<br>z=0.784<br>Cl1 2b (0.000, 0.000, 0.500)<br>Cl2 4e (0.000, 0.000, z)<br>z= 0.646
	K_5Cl_4	100-255	*I*4/*mmm*	*a* = 2.789 Å<br>*c* = 23.531 Å<br>(200 GPa)	K1 2a (0.500, 0.500, 0.500)<br>K2 4e (0.500, 0.500, z)<br>z = 0.612<br>K3 4e (0.500, 0.500, z)<br>z=0.276<br>Cl1 4e (0.000, 0.000, z)<br>z=0.669<br>Cl2 4e (0.000, 0.000, z)

					z=0.557
Metallic layered fcc-superstructure	K_3Cl	149-300	*I*4/*mmm*	*a* = 3.427 Å<br>*c* = 6.714 Å<br>(200 GPa)	K1 2b (0.500,0.500,0.000)<br>K2 4d (0.500,0.000,0.250)<br>Cl 2a (0.000,0.000,0.000)

Supplementary Table S2. Structures of B1-KCl and $P31m$-KCl_3 at 1 atm, $Pnma$-KCl_3 at 5GPa, $P\bar{3}c1$-KCl_3 and B2-KCl at 20 GPa, A15-type ($Pm\bar{3}n$) KCl_3, $Pm3$-KCl_7, $P\bar{4}m2$-K_3Cl_5, $I4/mmm$-K_3Cl and $I4_1/amd$-KCl at 240 GPa, and the corresponding atomic Bader charges (Q) and volumes (V).

	Lattice Parameters		X	y	Z	Q, |e|	V, Å^3
B1-KCl	a = 3.192 Å	K(4b)	0.500	0.500	0.500	+0.843	22.88
		Cl(4a)	0.000	0.000	0.000	-0.843	42.17
B2-KCl	a = 3.350 Å	K(1a)	0.000	0.000	0.000	+0.784	15.23
		Cl(1b)	0.500	0.500	0.500	-0.784	22.38
$Pnma$-KCl_3	a = 8.708 Å<br>b = 5.427 Å<br>c = 7.947 Å	K(4c)	0.827	0.750	0.536	+0.835	18.63
		Cl(4c)	0.933	0.250	0.769	-0.279	25.22
		Cl(4c)	0.162	0.750	0.640	-0.514	26.72
		Cl(4c)	0.878	0.750	0.052	-0.042	23.31
$P31m$-KCl_3	a = 8.587 Å<br>c = 6.206 Å	K(2b)	0.333	0.667	0.000	+0.878	23.43
		K(1a)	0.000	0.000	0.504	+0.876	25.13
		Cl(3c)	0.667	0.667	0.251	-0.404	37.40
		Cl(3c)	0.000	0.512	0.528	-0.049	33.20
		Cl(3c)	0.000	0.693	0.805	-0.424	37.52
$P\bar{3}c1$-KCl_3	a = 7.347 Å<br>c = 9.059 Å	K(2b)	0.000	0.000	0.000	+0.780	13.71
		K(4d)	0.333	0.667	0.143	+0.794	14.53
		Cl(6f)	0.260	0.260	0.250	-0.073	17.66
		Cl(12g)	0.289	0.403	0.897	-0.358	19.33
$Pm\bar{3}n$-KCl_3	a = 4.169 Å	K(2a)	0.000	0.000	0.000	+0.519	7.91
		Cl(6c)	0.000	0.500	0.250	-0.173	9.43
$Pm3$-KCl_7	a = 4.123 Å	K(1a)	0.000	0.000	0.000	+0.493	7.77
		Cl(1b)	0.500	0.500	0.500	+0.063	8.47
		Cl(6g)	0.745	0.500	0.000	-0.089	8.99
$P\bar{4}m2$-K_3Cl_5	a = 4.136 Å<br>c = 4.361 Å	K(1c)	0.500	0.500	0.500	+0.566	8.47

		K(2g)	0.500	0.000	0.245	+0.542	8.18
		Cl(1b)	0.500	0.500	0.000	-0.512	10.11
		Cl(4j)	0.252	0.000	0.747	-0.285	9.91
*I*4$_1$/*amd* -KCl	*a* = 3.340 Å<br>*c* = 6.873 Å	K(4a)	0.000	0.250	0.875	+0.545	8.595
		Cl(4b)	0.000	0.750	0.625	-0.545	10.579
*I*4/*mmm*-K_3Cl	*a* = 3.365 Å<br>*c* = 6.583 Å	K(2b)	0.500	0.500	0.000	+0.288	8.818
		K(4d)	0.500	0.000	0.250	+0.249	8.863
		Cl(2a)	0.000	0.000	0.000	-0.786	10.726
*I*4/*mmm*-K_2Cl	*a*= 2.749 Å<br>*c* = 7.587 Å	K(4e)	0.000	0.000	0.330	+0.344	9.19
		Cl(2a)	0.000	0.000	0.000	-0.688	11.30
*I*4/*mmm*-K_3Cl_2	*a* = 2.801 Å<br>*c* = 12.806 Å	K(2b)	0.500	0.500	0.000	+0.489	9.24
		K(4e)	0.500	0.500	0.798	+0.405	9.12
		Cl(4e)	0.000	0.000	0.897	-0.655	11.39
*I*4/*mmm*-K_4Cl_3	*a* = 2.791 Å<br>*c* = 18.190 Å	K(4e)	0.000	0.000	0.784	+0.413	9.13
		K(4e)	0.000	0.000	0.072	+0.449	9.17
		Cl(2b)	0.000	0.000	0.500	-0.673	11.66
		Cl(4e)	0.000	0.000	0.646	-0.621	11.31
*I*4/*mmm*-K_5Cl_4	*a* = 2.789 Å<br>*c* = 23.531 Å	K(2a)	0.000	0.000	0.000	+0.569	9.19
		K(4e)	0.500	0.500	0.612	+0.563	9.13
		K(4e)	0.500	0.500	0.276	+0.410	9.12
		Cl(4e)	0.000	0.000	0.669	-0.622	11.32
		Cl(4e)	0.000	0.000	0.557	-0.636	11.58
